# Acquisition and usage of robotic surgical data for machine learning analysis

**DOI:** 10.1007/s00464-023-10214-7

**Published:** 2023-06-30

**Authors:** Nasseh Hashemi, Morten Bo Søndergaard Svendsen, Flemming Bjerrum, Sten Rasmussen, Martin G. Tolsgaard, Mikkel Lønborg Friis

**Affiliations:** 1 Department of Clinical Medicine, Aalborg University Hospital, Aalborg, Denmark; 2 Nordsim—Centre for Skills Training and Simulation, Aalborg, Denmark; 3 ROCnord—Robot Centre, Aalborg University Hospital, Aalborg, Denmark; 4 Copenhagen Academy for Medical Education and Simulation, Center for Human Resources and Education, Copenhagen, Denmark; 5 Department of Computer Science, University of Copenhagen, Copenhagen, Denmark; 6 Department of Gastrointestinal and Hepatic Diseases, Copenhagen University Hospital - Herlev and Gentofte, Herlev, Denmark; 7 Department of Urology, Aalborg University Hospital, Aalborg, Denmark

**Keywords:** Robotic surgery, Artificial intelligence, Data acquisition

## Abstract

**Background:**

The increasing use of robot-assisted surgery (RAS) has led to the need for new methods of assessing whether new surgeons are qualified to perform RAS, without the resource-demanding process of having expert surgeons do the assessment.

Computer-based automation and artificial intelligence (AI) are seen as promising alternatives to expert-based surgical assessment. However, no standard protocols or methods for preparing data and implementing AI are available for clinicians. This may be among the reasons for the impediment to the use of AI in the clinical setting.

**Method:**

We tested our method on porcine models with both the da Vinci Si and the da Vinci Xi. We sought to capture raw video data from the surgical robots and 3D movement data from the surgeons and prepared the data for the use in AI by a structured guide to acquire and prepare video data using the following steps: ‘Capturing image data from the surgical robot’, ‘Extracting event data’, ‘Capturing movement data of the surgeon’, ‘Annotation of image data’.

**Results:**

15 participant (11 novices and 4 experienced) performed 10 different intraabdominal RAS procedures. Using this method we captured 188 videos (94 from the surgical robot, and 94 corresponding movement videos of the surgeons’ arms and hands). Event data, movement data, and labels were extracted from the raw material and prepared for use in AI.

**Conclusion:**

With our described methods, we could collect, prepare, and annotate images, events, and motion data from surgical robotic systems in preparation for its use in AI.

Robot-assisted surgery (RAS) is the latest advancement in clinical minimally invasive surgery (MIS). RAS has improved surgeons' dexterity, precision, and ergonomics, and has made complex surgical procedures easier compared to other types of MIS [1, 2]. RAS has also contributed to improvements in surgical outcomes such as fewer peri- and postoperative complications in different surgical fields [3, 4].

Yet, patient outcomes remain directly associated with surgical performance [5–8]. Insufficient training and poor technical skills can compromise the clinical outcome, and increase rates of readmission, reoperation, and overall morbidity and mortality [5–8]. Various assessment tools, such as the Global Evaluative Assessment of Robotic Skills (GEARS), have been developed for the assessment of robotic skills [9, 10].

Existing assessment methods are expert-based, time-consuming, and demand large resources [7–11]. In recent years, computer-based automation of the assessment of surgical skills has been seen as a promising alternative to expert-based assessments [12–16]. Among these, technologies based on artificial intelligence (AI) have been proposed to improve the affordability of continued assessments, reduce bias assessment costs, reduce rater bias, and improve the reliability of skills assessments [14, 17–19].

Despite these advantages, there are multiple unsolved challenges relating to the usability of AI technology for surgical skills assessment [20]. Most importantly, the use of AI relies on high-quality data, but available datasets have so far been lacking in quantity and quality [17, 18, 20]. There is a lack of standard protocols for data capture, data preparation, and annotation before using data in AI algorithms, which may also impede the implementation of AI in surgery [20, 21]. Although commercial systems have been described such as the dVLogger [9], issues with ownership of data, General Data Protection Regulation (GDPR) obstacles and the fact that these systems are only accessible on a permission-based practice make them unsustainable for future data capture. Finally, there is a need to ensure standardized methods for data capture and preparation before implementing the data in AI algorithms irrespective of the type of robotic system used, which has not yet been described.

We present a method to collect image and motion data from a surgical robot and how to prepare data for subsequent machine learning algorithms. This is an attempt to describe a method, making it practical, in order to ease capturing, preparation, and annotation of images and motion data before using it for AI development.

## Materials and methods

Our proposed method for data acquisition and annotation follows a series of steps during and after data collection:

1. Capturing image data from the surgical robot
2. Extracting event data
3. Capturing movement data of the surgeon
4. Annotation of image data

We describe this process below drawing on examples from previous research, practices from other domains, and previous experiences with data capture systems for a broad range of devices, including laparoscopy, endoscopy, colonoscopy, and RAS [14, 22–24].

### Setting, equipment and participants

The da Vinci Surgical System (dVSS) is a robotic telesurgical system, where the surgeon controls the robot instruments remotely [25]. So far, there have been five generations of dVSS; da Vinci Classic, da Vinci S, da Vinci Si, da Vinci X, and da Vinci Xi. The basic concept has remained the same in all generations, however, the platform has improved with each model [26]. All systems comprise a surgeon’s cart, where the surgeon sits; a patient cart, where the instruments are fixed; and a vision cart that links all components together [26].

We have tested our method on porcine models with both the da Vinci Si and the da Vinci Xi, with some minor adjustments and differences in the outcome, as will be described in the next sections. The tests were carried out in the Biomedical Laboratory at Aalborg University Hospital, with the approval of The Animal Experiments Inspectorate under the administration of Danish Veterinary and Food Administration, ID: 2018–15-0201–01392.

Based on prior works regarding learning curves for RAS, we defined two groups of participants. Novices who have performed under 100 RAS procedures, and experienced who have performed more than 100 RAS procedures [27, 28]. The participants were included in the study in relation to their participation in RAS-courses at the Biomedical Laboratory at Aalborg University Hospital.

Statistical analysis was done using Stata, Stata/MP 17 (2 cores), StataCorp LLC. Mean and standard deviation (SD) was used to report event data.

### Step 1. Capturing image data from the surgical robot

Prior studies in the field of AI and RAS have used the dVLogger to capture image and system data from the dVSS or used datasets that were made in collaboration with Intuitive Surgical Systems Inc [5, 9, 13, 20]. The dVLogger is a software device that records video in endoscopic view with 30 frames per second (FPS), system data such as kinematic data (instrument movement, instrument travel time, velocity, path length) and event data (frequency of clutch use, third arm swap, camera movement, and energy use) in 50 Hz through an Ethernet connection [9].

Our method will seek to capture raw image data of the surgical system from the surgeon’s console (robot control platform).

**Fig. 1:**
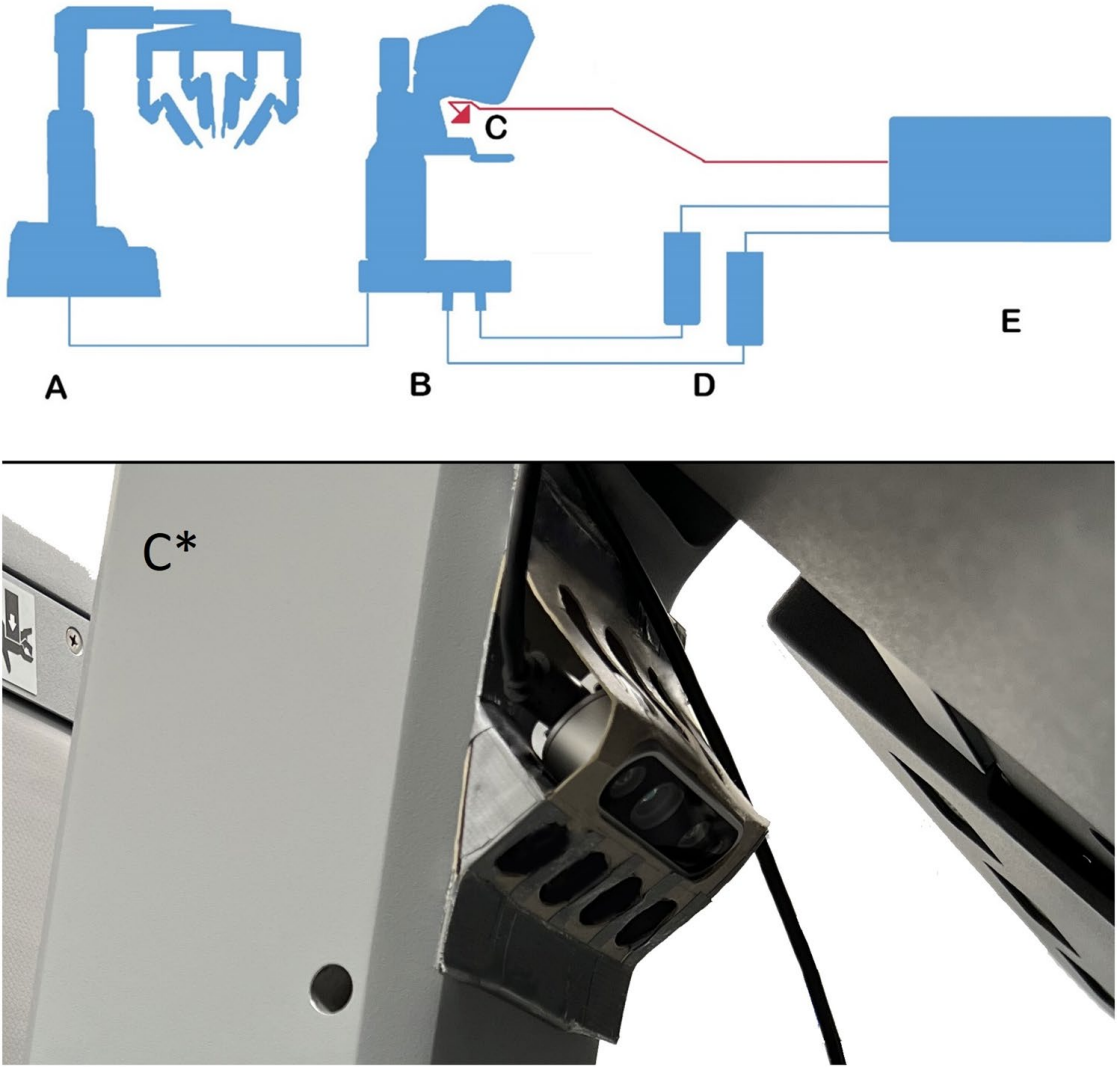
Data collection from da Vinci Surgical System and depth cameras. **A** Image outputs from the surgical robot **B** The surgical console used by the surgeon. **C** Depth camera capturing 3D footage of surgeon’s movements. **D** Capture devices used as a gateway to capture image output. The capture device is connected through a DVI to HDMI converter. **E** Local computer for recording and storage of footage. C* shows the cardboard camera holder

Since the dVSS uses a stereoscopic camera with two oculars, raw video footage of the right and left endoscope ocular can be accessed through the back of the surgeon’s console using two HDMI to USB Video Capture Cards (VCCS), one for each ocular output. Many VCCS exists (Maxwell, Epiphan, etc.), and most are equally good, our previous choice of VCCS have been dependent on type of video signal, i.e., SDI or HDMI, or based on the operating system of the recording PCs. In this case we used HDMI to USB video capture card from Ozvavzk. Both capturing cards should be connected to a computer through two different USB slots, best not to use a USB hub. For physical connection between the capture cards and the robot, we used two HDMI cables connected to the robot through two HDMI-to-DVI Cables (0.15 m HDMI Male to DVI Female). The setup is illustrated in Fig. 1. The image output from the robot were recorded using open-source software (Open Broadcaster Software Studio, Wizards of OBS, OBS Studio v. 27.2.4, 64-bit), which allows for multiple inputs to be displayed and recorded at synchronously.

Resolution and FPS can be configured in OBS studio before recording. In this study, 2560 × 720 was used with 15 FPS. For the sake of simplicity, only one of the endoscopic ocular outputs was used in this study, using two however could enable 3D data analysis of the field, using stereogrammetry. The video was cropped in separate left and right views using Free Video Crop, RZSoft Technology Co. Ltd, v. 1.08, to 1280 × 720 format. Whenever sequences were recorded where surgical instructions were given during the RAS courses or changes of instruments took place where nothing of surgical relevance occurred in the video footage, these sequences were cut out using Windows Video Editor, Microsoft Corporation. Other free video editors and recorders are also available such as the command-line software called FFmpeg, which was also used in this study.

**Fig. 2:**
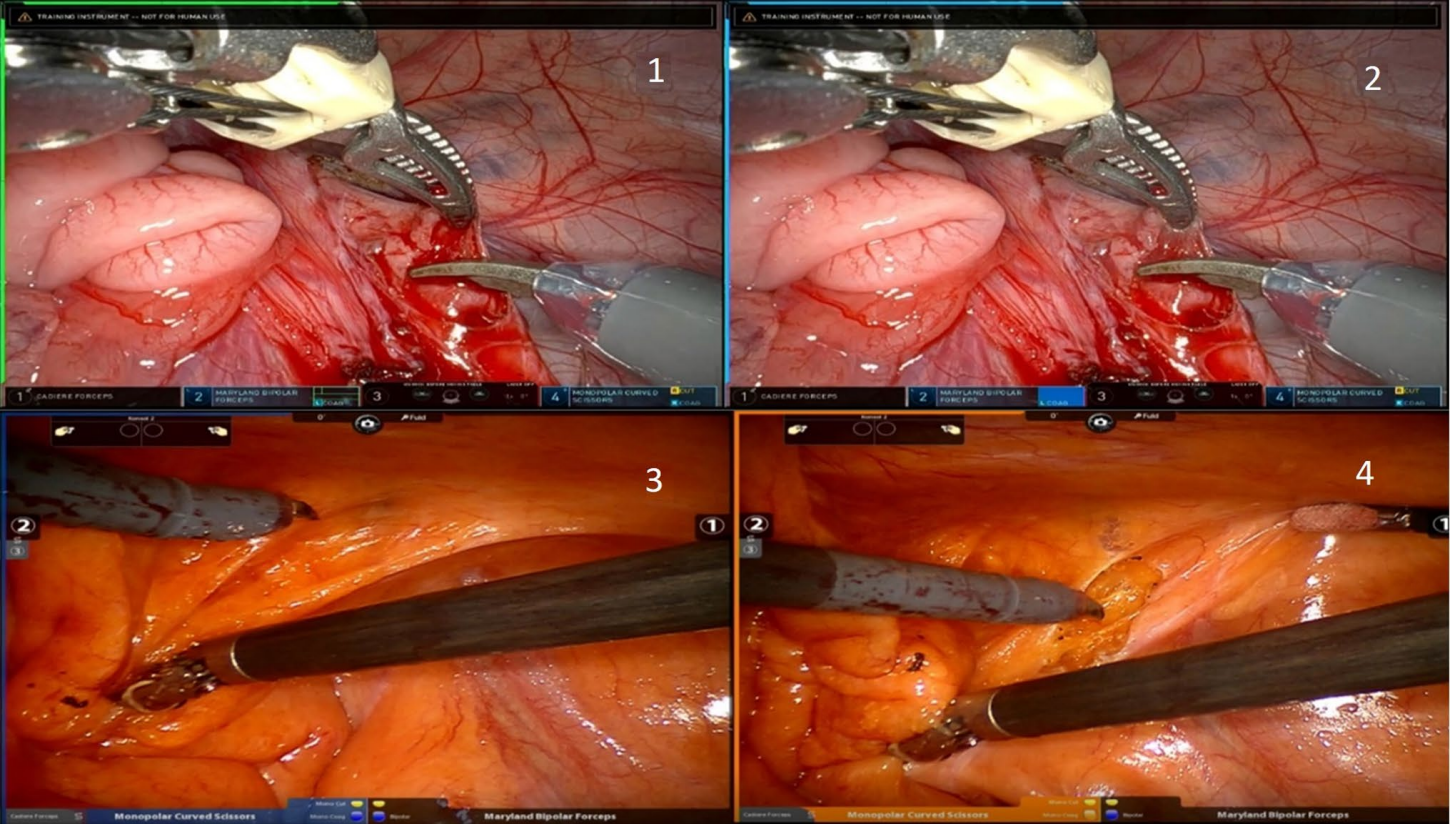
The lines of readiness and activation of the instruments. In picture 1 the green horizontal and vertical line at the top and left side indicates that the surgeon’s foot is hovering above the coagulation-pedal which is pressed in picture 2. In pictures 3 and 4 the same is seen for the left cut-pedal of the da Vinci Si system

Connecting VCCS the computer was the same for both the da Vinci Si and the da Vinci Xi, only difference being in the video output, as seen in Fig. 2. There were mainly differences in the on-screen placement of bars and indicators and are described in further detail in the upcoming sections.

### Step 2. Extracting event data

From the raw image data event data such as the use of cut and coagulation, use of the clutch, third arm swap, and camera movement can be attained. Camera movement being the time slots where the camera is activated and used. The image data presents all the mentioned features on screen in the four panels displayed at the bottom of the video screen, see Fig. 2.

#### Use of cut and coagulation

The cut and coagulation functions can be activated for both arms on the da Vinci Xi system, depending on the instruments used during the procedure. In the extracted raw image data, green lines will show at the sides of the picture, whenever the surgeon’s foot hovers above the pedal, which activates cut or coagulation, respectively. This happens through the pedal sensors of the surgical console. When the pedal is pressed the lines will change color to yellow for cut and blue for coagulation. The lines will show on that side of the picture, which indicates the activated instrument. When the right instrument is activated, only the right side of the screen will have colored lines and vice versa, see Fig. 2.

Figure 2 also demonstrates how the on-screen cut and coagulation symbols in the panels at the bottom of the screen will also change color to yellow or blue when activated.

In the older da Vinci Si system, the same principles are applicable, however, there are no color differences when using cut/coagulation. When the surgeon’s foot hovers above the pedal, blue lines will show on the respective side of the screen. When pressed, the lines will change color to orange, and so will the symbols in the middle of the screen representing the cut and coagulations pedal. An example of this is shown in Fig. 2.

#### Use of clutch

**Fig. 3:**
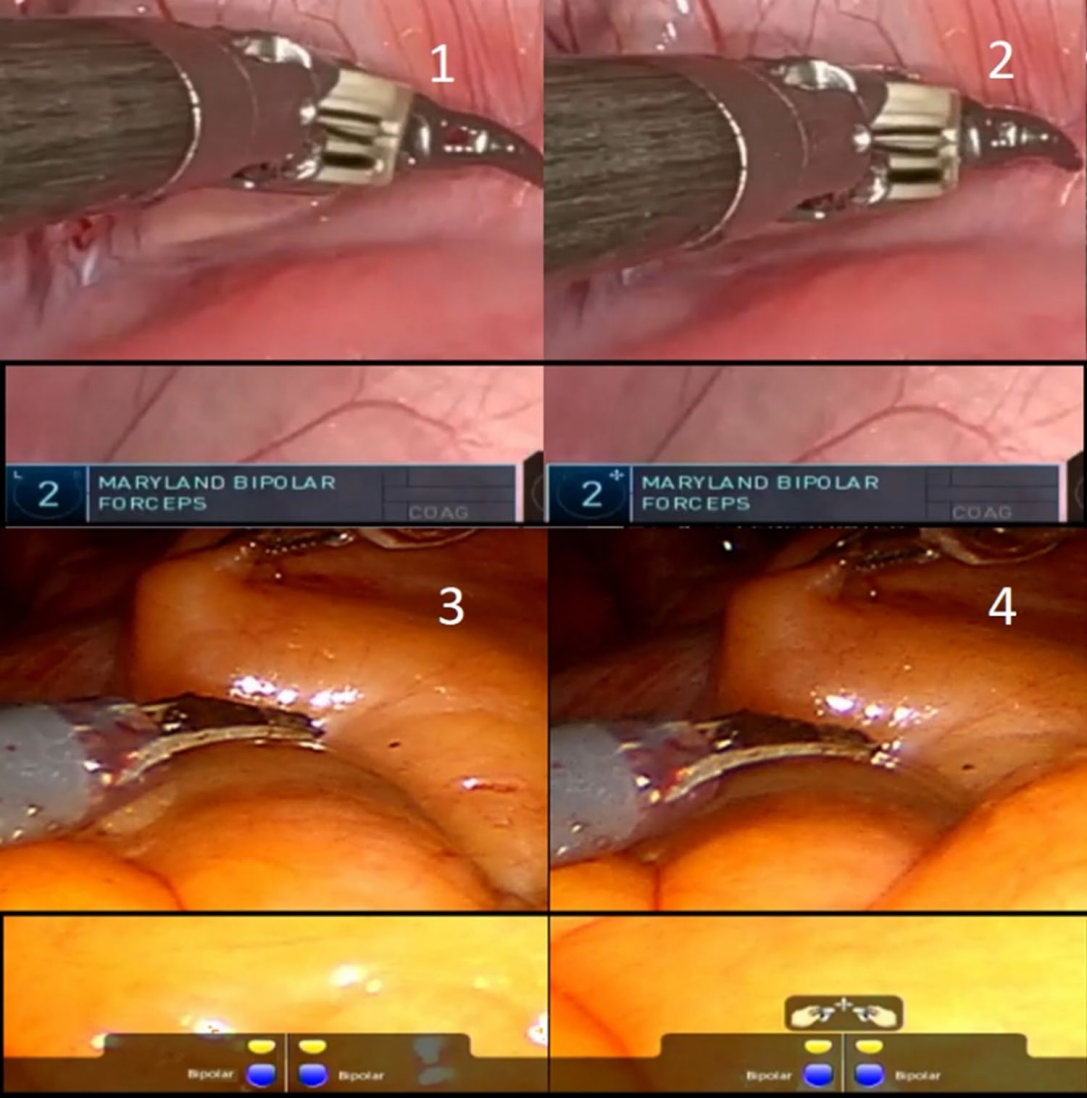
The use of clutch function and change of symbol in the panel at the bottom of the raw video. The change can be seen at the number two, shifting from a letter indicating the Left-side to a four-sided arrow. Picture 3 and 4 shows the clutch symbol of the da Vinci Si system. The symbol appears in the middle of the bottom part of the screen

Every time the clutch is used in the da Vinci Xi surgical console to lock the instruments and position the hands, the panels of the active arms will show a shift from the sign indicating the active instruments (left and right, respectively) to a four-sided arrow indicating clutch use, see Fig. 3.

In the da Vinci Si system, the use of a clutch is shown in the bottom part of the screen, with a similar four-sided arrow as seen in the da Vinci Xi system, see Fig. 3. It is shown as a single symbol representing both left- and right-hand clutch because the da Vinci Si system only allows for conjoined clutching of the controllers.

#### Third arm swap

**Fig. 4:**
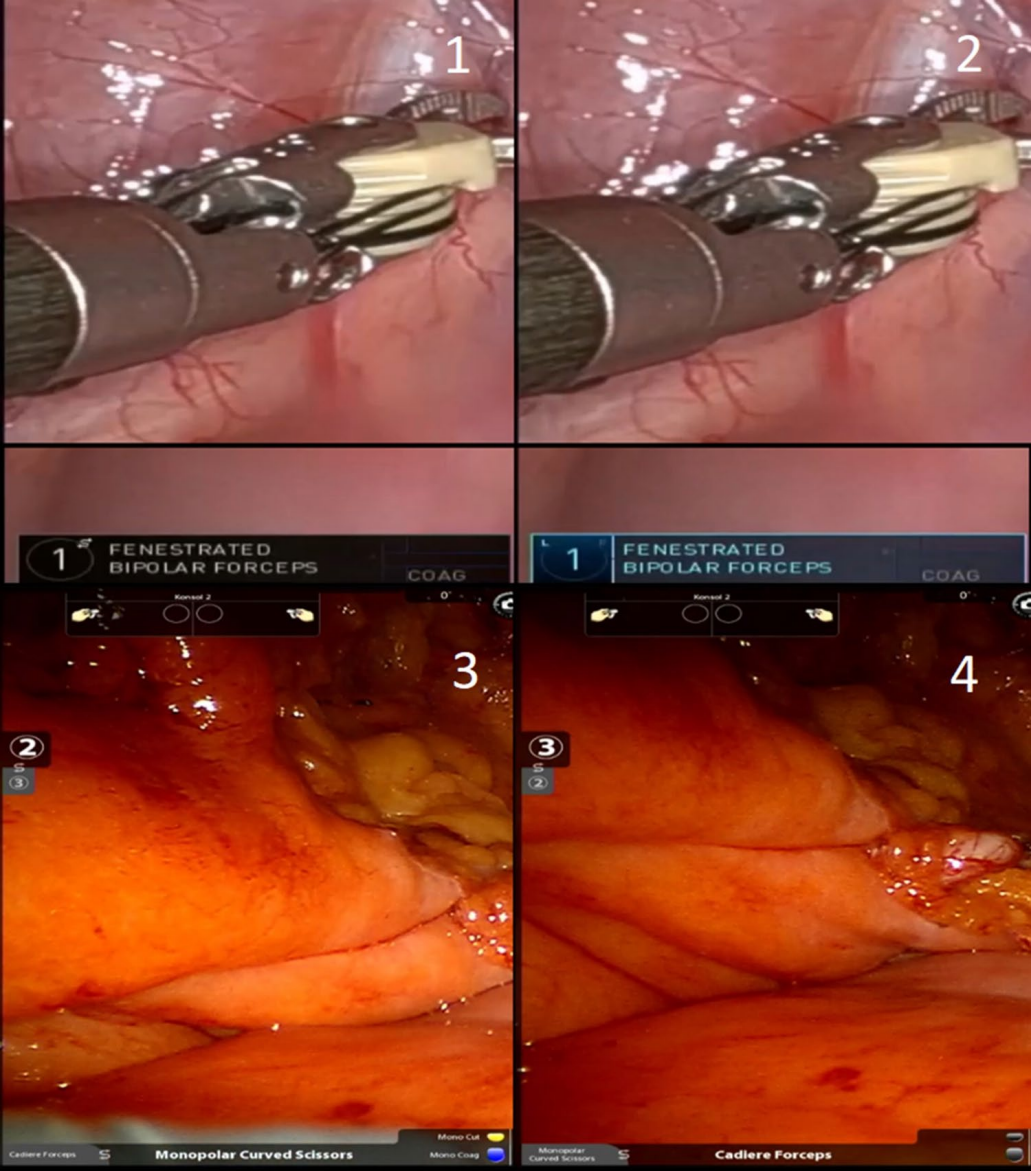
The activation of the third-arm and change in color and symbol. The swap symbol seen in the first picture disappears and the color is turning blue when the arm is swapped and activated. Picture 3 and 4 shows the indicators for third arm swap in the da Vinci Si system

The third arm in the dVSS refers to the extra robotic arm which is mostly used to assist in holding tissue or when another instrument is needed. It can be activated in the da Vinci Xi when using the side pedal to swap from one of the main arms to the third arm. Every time the swap is made the panel of the third arm, or the arm that is being activated, will change color and the swap symbol will disappear from the panel, as seen in Fig. 4.

The third arm swap is activated the same way in the da Vinci Si, as in the newer da Vinci Xi. However, when activated, an indicator on the left side, or right side, of the screen tells which robot arm is being swapped, and the panel at the bottom of the screen will show which instrument is becoming the main instrument of the arm, see Fig. 4.

#### Camera movement

**Fig. 5:**
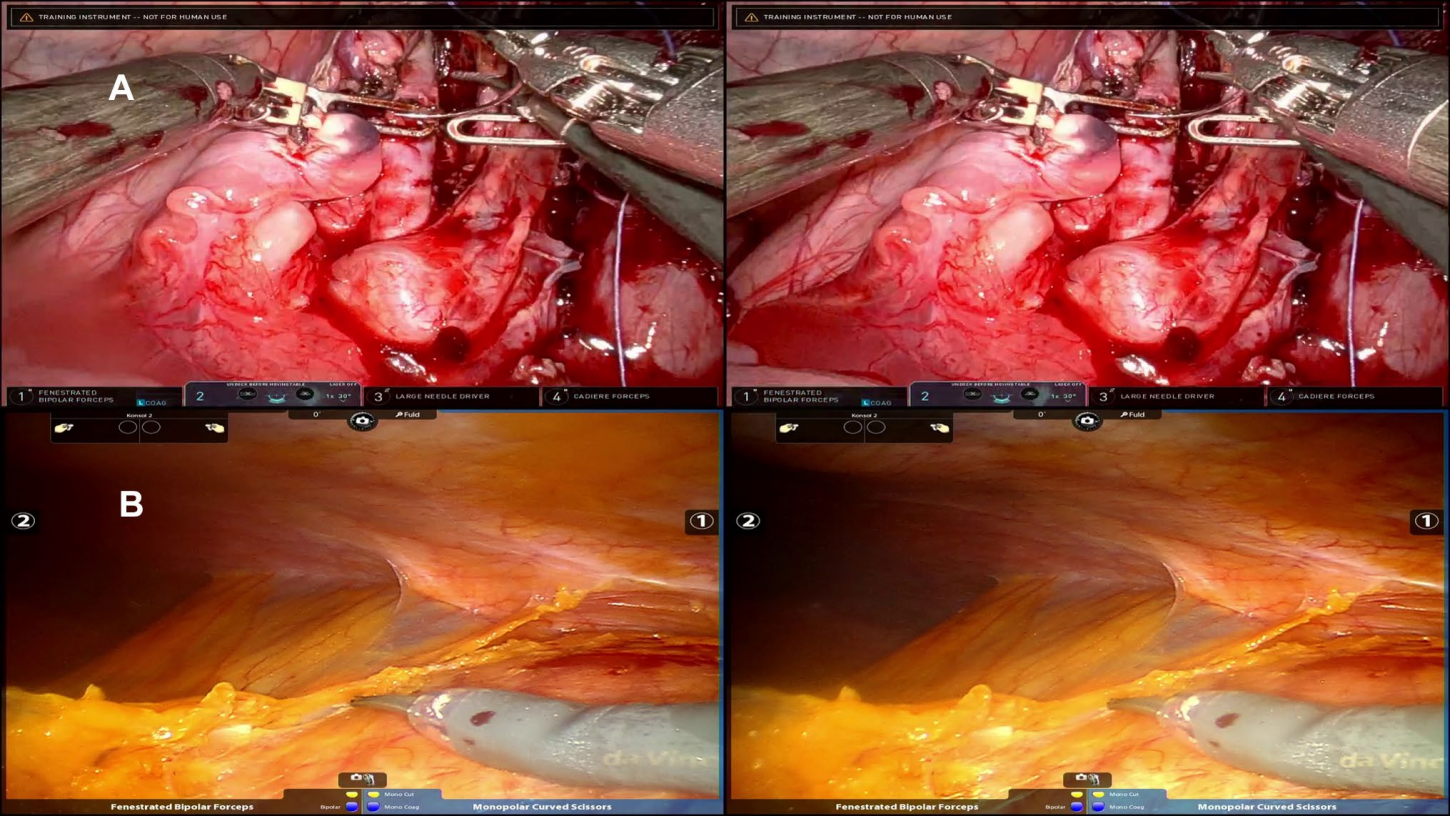
The raw video file recorded from the surgeon’s console of the da Vinci Surgical System on porcine model. Both left and right endoscopic ocular are presented in the sample. Pictures A is from the da Vinci Xi, and the picture B is the da Vinci Si. In both pictures the camera movement is activated on both systems

When the surgeon wants to move the camera for a better angle of view in the da Vinci Xi, the camera pedal is pressed. When the camera movement is activated the on-screen camera panel at the bottom of the screen changes color, as seen in Fig. 5.

Besides the activation of the camera, information about the tilt of the camera is also shown on the camera panel.

In the da Vinci Si system, the camera movement is activated similarly to the da Vinci Xi. However, it will show a camera symbol at the bottom part of the screen. The camera tilt is always visible at the top part of the screen.

#### Extraction of event data

Event data can be manually recorded/extracted in excel by counting every event from the videos. However, it can also be automatically detected using the open-source computer vision library, OpenCV, which includes algorithms for computer vision. The first author hosts active repositories on Github with example code for parsing and interpreting the data type available at www.Github.com/NasHas [29].

The example code scans every frame of a video, logs the region of interest (ROI), which are the indicators for clutch, coagulation etc., and registers the count each time the defined target pattern shows on the screen. An accuracy threshold must be set for each pattern which indicates how sensitive the pattern recognition is towards it. We used a threshold of 0.95 for coagulation and clutch and a threshold of 0.90 for camera movement and third arm swap (1 being the maximum value). It is important that the target patterns are of the same resolution as the video files, otherwise the algorithm will not respond.

Event data can be tabulated as ‘total amount of usage’ and/or ‘uses per minute’. Previous studies have used event data as an additional indicator of the level of expertise, and an extra variable in machine learning algorithms [7–9, 12].

### Step 3. Capturing movement data of the surgeon

**Fig. 6:**
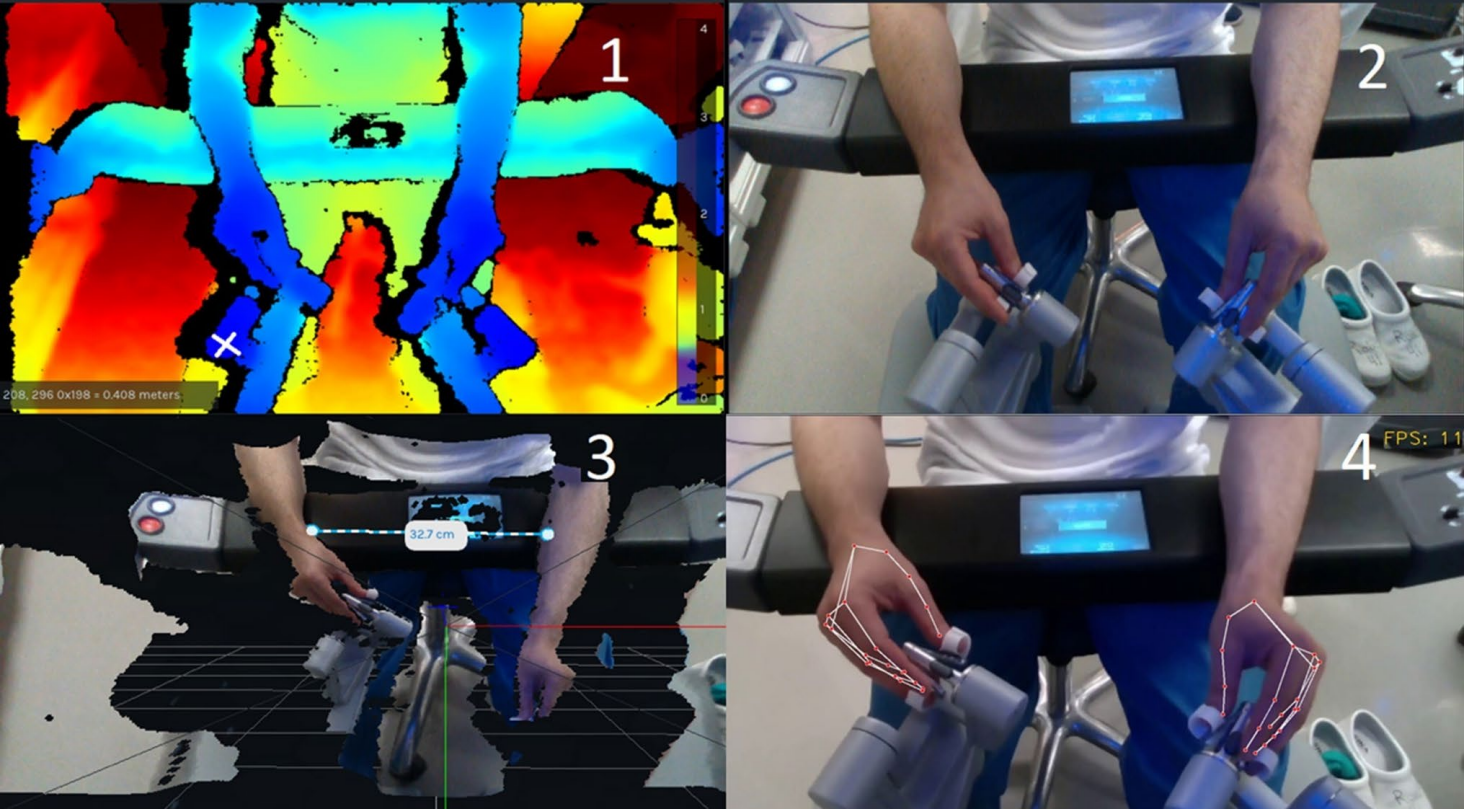
Depiction of the output from the 3D camera. (1) shows the depth module of the 3D camera. For every point of the picture, a coordinate and a distance from the camera are given. (2) shows the normal 2D module. (3) shows the 3D module of the Intel Realsense Software, where precise measurements can be made. (4) shows hand-tracking software that tracks the movement of hands

The movement data is represented through 3D footage of the surgeon’s arms and hands. See Fig. 6.

It is captured using a 3D motion/depth camera, Intel RealSense Depth Camera D435i, mounted in a cardboard holder at the surgeon’s console, as seen in Fig. 1C*. The motion capture camera should be placed in such a way that all movements from the elbow to the hands are visible in the captured footage. Capturing and processing of the 3D motion camera can be made using an open-source software development kit (SDK) from Intel’s official webpage or from their Github page (Intel® RealSense™ Viewer SDK 2.0 (v2.50.0) www.Github.com/IntelRealSense). All motion and depth recordings can be compressed to reduce file sizes.

The Intel Realsense software saves data in BAG-format, but recorded footage can be converted using the convert tools of the software to other file formats such as; PNG, CSV, RAW, PLY, and BIN. We present a Python example which converts the BAG-files to mp4-format available at www.Github.com/NasHas [30].

If kinematic data is needed such as the path length of the movements of the hands, data can either be analyzed using the RealSense SDK or analyzed in different object tracking softwares or scripts. Examples of software libraries are OpenCV, MediaPipe, or Simple Online and Realtime Tracking (SORT) [31], which tracks defined objects in real time. Path lengths can be found using path tracking software making kinematic data available from the image data recorded from the 3D camera. In this study, we exemplified this using OpenCV and AI based MediaPipe Hands library for hand tracking available at www.Github.com/NasHas [32].

Our solution first finds landmarks of the hands, then tracks and calculates the path lengths in pixels and allocates a color for both hands. Different landmarks can be specified and tracked, in this study, we tracked the wrists, but fingers and other parts of the body such as elbows are available in their models for tracking.

### Step 4: Annotation of image data

After the capture and preparation of image data, endoscopic video data, and movement video data, as described in the abovementioned sections, the last step before implementing the data in a machine learning algorithm is to annotate, or label, the image data so it can be used to correctly train machine learning algorithms. In general, two ways of annotations can be made when dealing with image data either temporal labeling of the time frames of events in a video sequence or visually labeling spatial elements in the field of view [13, 20, 21].

In this study, temporal labelling is used as an example, where labels are made in three general categories, representing the basic elements of surgery,’suturing’, ‘dissection’, and ‘other’ (being events such as suction or holding) [33, 34]. Under each category, subcategories are defined, which can either be analyzed singlehandedly or seen as a whole, as all the subcategories make up the main category, see Table 5. These general categories and subcategories may be defined as fits best depending on the type of features of interest for the machine learning algorithm.

Different types of publicly available software can be used to label temporally, some more difficult to use than others. In this study, the Behavioral Observation Research Interactive Software (BORIS, v. 7.13.8)[35], which can be downloaded from their official webpage or their Github, was used and found to be user-friendly (www.Github.com/olivierfriard). It was originally made to observe animal behavior and label time stamps in concordance with events of video footage. BORIS makes it easy to define the categories and subcategories, in general ‘events’ of the video, assigning all of them a keyboard key to easily start and stop the label according to the occurrence of an event. The labels can then be exported in TSV text files, in which each video name, label, and time stamp are noted along with the definition table of all labels. BORIS generates columns in each document, of which three output columns are of relevance for later use in machine learning algorithms: time, behavior, and status. The time column represents the time of change in behavior, the behavior column is the classification of the label in its respective subcategory, and the status column marks when a behavior starts/stops at its given time. This makes it easy to integrate with a machine learning algorithm that can then use the labeled time stamps to extract the sequences of interest from each video recording. All labels were annotated manually by a urological resident doctor (NH).

When labeling spatial elements of a video sequence there are also many different possibilities available on Github. A tool that is often used is the Visual Object Tagging Tool (VOTT) which can label image and video frames and export labels to be used in a machine learning algorithms [21]. Spatial labelling was not exemplified in our case to maintain brevity and relevance. But could include marking the instruments in view, enabling better tracking, or highlighting anatomical landmarks to recognize them as events in the context of analysis the surgical process.

## Results

We included data from 15 participants (11 novices and 4 experienced RAS surgeons), see Tables 1 and 2. In total, 10 different intraabdominal RAS procedures were performed on porcine models, depending on the RAS-course that was conducted in the Biomedical Laboratory. A total of 188 videos of various lengths were recorded. 94 videos from the surgical robots, endoscopic footage, and the corresponding 94 videos of the surgeons’ hand movements using the 3D camera, as seen in Table 3.

**Table 1:** Participant demographics

	Novices	Experienced
Number of participants	11	4
Sex (Men/Women)	4/7	3/1
Age (mean (SD))	38.2 (7.0)	47 (10.9)
Handedness (L/R)	2/9	1/3
Occupation & specialty		
Resident/Registrar	9	
Specialist doctor	2	4
Urology	8	4
Gynecology/Obstetrics	2	
Thoracic surgery	1	
Robotic surgical experience (total cases)		
< 50	11	
> 200		2
> 400		2

*L/R* Left/Right, *SD* standard deviation

**Table 2:** Overview of procedures and number of recordings made of the participants

	Number of participants	11	4
Number	Procedure	Novices	Experienced
1	Salpingectomy (fallopian)	12	2
2	Bladder puncture	16	3
3	Lymph node dissection	20	8
4	Partial nephrectomy	4	2
5	Nephrectomy	2	1
6	Ureter dissection	11	
7	Ureter implantation	5	
8	Ureter anastomosis	2	
9	Cystectomy		1
10	Bowel puncture	2	3
	Sum of recordings	74	20
	Total recordings	94

**Table 3:** Technical overview of the video footage extracted from the surgical robot and the 3D footage of the surgeons’ arm movements from the Intel Realsense 3D camera

	Video footage	3D footage
Total time (minutes & seconds)	1447 min., 26 s	1450 min., 8 s
Total size (gigabytes)	72.91 gb	3947.61 gb
Width and length (pixels) (total amount)	848 × 240 px (21), 2560 × 720 px (73)	640 × 360 px (42), 848 × 480 px (31), 1280 × 720 (21)
Frames per seconds (FPS) (total amount)	30 FPS (21), 15 FPS (73)	30 FPS (21), 15 FPS (73)

### Image data, event data, and annotations

The raw image data from the video files contained both left and right ocular feeds side-by-side, see Fig. 5. After the initial extraction, the video files were cropped into a one-picture view, and event data was extracted, as seen in Table 4. Initially, 21 cases were captured using lower resolution and higher FPS, this however resulted in longer processing times during event data extraction. The following 73 cases were recorded with the same higher resolution and lower FPS (2560 × 720px and 15 FPS). For comparison, all the surgical procedures which were made by both the novices and experienced are presented with event data in Table 4. The computational processing times are also tabulated. Note that the cut-function is not tabulated. It was only used two times during lymph node dissection by a novice participant, and therefore not tabulated.

**Table 4:** Event data for all surgical procedures that had both experienced and novices represented

	Armswap (mean (SD))		Camera (mean (SD))		Clutch (mean (SD))		Coagulation (Right) (mean (SD))		Coagulation (Left) (mean (SD))		Duration (mm.ss) (mean (SD))		Total computer extraction time (hh.mm.ss) (mean)	
	Exp	Novice	Exp	Novice	Exp	Novice	Exp	Novice	Exp	Novice	Exp	Novice	Exp	Novice
Salpingectomy	2.5 (2.1)	9.4 (3.9)	26.5 (5.0)	38.9 (21.41)	7.5 (0.7)	19.4 (11.6)	22 (4.4)	31.1 (20.9)	2.5 (3.5)	4.8 (6.8)	05.29 (01.55)	23.02 (10.31)	00.33.32	01.29.48
Bladder puncture	1 (1.7)	4.9 (4.7)	14.3 (6.4)	19.1 (15.6)	4.3 (5.7)	17.9 (17.4)	4 (6.1)	9.1 (8.2)	3.3 (5.8)	0.9 (2.5)	05.50 (00.59)	19.24 (06.07)	00.37.08	02.18.10
Lymph node dissection	1.1 (1.3)	1.4 (2.1)	5 (4.2)	14.4 (11.0)	3.9 (5.2)	7.2 (6.6)	12.5 (12.1)	33 (25.3)	5.4 (10.3)	1.4 (3.6)	02.09 (02.20)	09.22 (03.49)	00.10.24	00.59.00
Nephrectomy	0	0 (0)	5	59 (17.0)	7	22 (11.3)	2	5.5 (3.5)	0	19.5 (27.6)	03.42	25.33 (15.55)	00.22.14	01.51.10
Partial nephrectomy	0 (0)	0 (0)	5 (1.4)	101.5 (29.8)	5 (1.4)	39.3 (29.8)	7 (8.5)	19.3 (20.7)	0.5 (0.7)	11 (22)	16.09 (07.55)	28.55 (20.39)	01.06.58	03.02.17
Bowel puncture	0 (0)	2 (2.8)	5 (4.2)	10.5 (5.0)	2.3 (2.5)	26.5 (10.6)	3 (4.4)	0 (0)	4.3 (7.5)	0 (0)	04.56 (01.25)	19.05 (06.18)	00.29.53	01.14.53

*Exp* experienced, *SD* standard deviation, *hh*.*mm*.*ss* hours.minutes.seconds

Note the last column describes the processing time to automatically extract event data

For each video, a corresponding document with temporal labels was made by using BORIS. All labels in each category and subcategory are presented in Table 5.

**Table 5:** Categories and subcategories of annotations used in the study

Class (categories)	Annotation (subcategory)	Total amount of labels	Total labels in each category
Suturing	Suture puncture	412	
Suturing	Suture handling	570	
Suturing	Suture, single	13	
Suturing	Suture, running	37	1032
Dissection	Dissection, ordinary	769	
Dissection	Dissection, clips	125	
Dissection	Dissection hemostasis	82	976
Other	Suction	46	
Other	Camera handling	1754	
Other	Changing instrument	34	
Other	Cleaning	7	
Other	Holding with 4. Arm	242	
Other	Holding with other instruments	2	
Other	External instrument (non-robot)	310	
Other	Catheter placement	4	2399

Note the total amount of labels in each category made by using BORIS

### Movement data

**Fig. 7:**
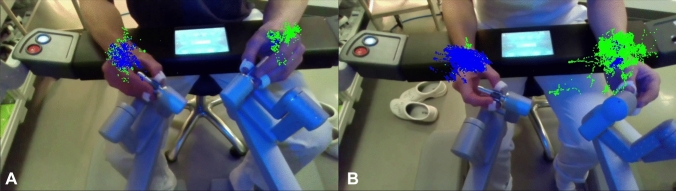
Comparison between hand movement paths of an experienced (**A**) and novice (**B**) during lymph node dissection. Path lengths are seen in Table 7. Pixels are placed at the wrists (blue for left and green for right) in each frame and connecting pixel between each point. Note the focused and shorter/less dense path of the experienced. Some dots are overlapping due to tracking errors when some hand-landmarks get out of the camera field of view

The data on the surgeon’s movement are raw image 2D footage of arm and hand movements, and depth footage of arm and hand movements (a 3D view can also be entered in the RealSense SDK where precise measurements of length are available) which can be used for visual analysis. Besides the visual analysis, the RealSense SDK also provides coordinates of each frame, which may be used for more in-depth numerical analysis. A sample of data output is illustrated in Fig. 6 and Table 6. As can be seen in Table 3 the raw 3D footage requires a large storage capacity. The data was captured while experimenting with different resolutions and FPS, eventually resulting in 640 × 360px and 15 FPS being the lowest settings enabling the initial analysis at expected acceptable precision. The total length of the recorded 3D data is longer, because starting and stopping times were synchronized with the surgical image data stream. We were able to extract video files of the surgeons’ movement, to create a movement path of the wrists and determine their path lengths, see Fig. 6.4, Fig. 7, and Table 7. Figure 7 shows a visual comparison between an expert and a novice surgeon. The path lengths are outputted in pixels.

**Table 6:** Example of extracted data from Intel Realsense Viewer with 2D pixel-coordinates and 3D length

2D	3D
160, 341	208, 296 0 × 198 = 0.408 m

**Table 7:** An example of path lengths of the wrist movement of surgeons using OpenCV and MediaPipe

	Experienced	Novices
Right wrist movement	8.5 M pixel	73.6 M pixels
Left wrist movement	19.3 M pixels	186.9 M pixels
Duration	48 s	6 min 25 s

The software detects the wrist and places a dot for every image. It then draws a line between each dot and calculates the path length in pixels. The duration of movement during the same procedure is also tabulated

## Discussion

In this paper, we present a method of capturing raw image data from the da Vinci Si and Xi systems using capturing devices and a 3D motion/depth camera. From the raw image data, we introduced a method of extracting event data and kinematics, which can later be used and analyzed in machine learning algorithms. We also present ways of annotating the prepared image data which creates ground truth labels for machine learning algorithm training.

Event data were extracted automatically, and only by one set of target patterns for all videos of the higher-resolution videos. However, in the initial lower-resolution videos, new target patterns needed to be made because of changes in resolution. Furthermore, every video file needed their own individual target patterns because the pixelated quality of the lower-resolution files made it difficult for the algorithm to match the target patterns with the video frames. It also resulted in longer processing times and adjustments in threshold value for each lower-resolution file.

Categories of event data and kinematic data can be extracted and used for building machine learning algorithms. The dVLogger from Intuitive provides data that can be filtered to exclude clutching by the surgeons when tracking the movements of the controllers of the surgical console [9]. This cannot be directly achieved using the current method, however the basis for further development of the method has been laid, and the approach is likely to work on other RAS systems as well. It may be solved by annotating all clutching from the surgical footage and from the 3D camera footage, and afterward excluding clutching points from tracking. Although time consuming, when done manually, it can be done automatically, as presented in the current paper. Another way of tracking movement could also be to only record tracking of the instruments in the surgical endoscopic footage. Although all annotations can be synchronized, but as in this example both surgical footage and footage from the 3D camera must be annotated separately with the same labels. If using events that can be identified on both data streams, then synchronization is enabled, e.g., initiating recording on both, and make sure both are recording the same physical space, e.g., the console, then an event could be introduced with something like a Clapperboard or clapping hands. The event being the exact frame in both streams where the hands meet. This is a disadvantage compared to the dVlogger. However, advantages include broad availability of the recording system, that one can easily determine if there is a shift of surgeon during a procedure, and standardization of recordings across RAS systems, all which are not descriptive of the dVlogger.

Because the dVlogger records the coordinates of the instruments, the movements of instruments, and indirectly the movement of surgeons, can always be tracked, no matter where they might be in the field of motion [9]. When using a 3D camera, with a fixed field of vision, or using the endoscopic camera footage, there may be spots where one hand or part of the hands, or an instrument or part of the instrument, may be out of the field of view. This is illustrated in Fig. 7, where the left hand is represented with blue color and the right hand with green. Some dots are seen overlapping because, when the hands of the surgeon get out of the field of view, the algorithm registers movement from that hand in place of the remaining hand. However, newer AI-based tracking algorithms as mentioned in previous sections, can make pose estimations based on several selected landmark points of the body, instead of only one. If only part of the hand is out of the field of vision, the rest of the hand can have enough landmark points to remain trackable. If all the hand is out of the field, a landmark point based on the wrist can be used to continue movement tracking. Also, the current method uses 2D footage as proof of concept. Furthermore, the output files of the 3D footage were large and needed much computer power, as seen in Table 3, making 3D analyses more demanding, and beyond the scope of this description. By always compressing the files, using less resolution and FPS, we were able to reduce the file size, without compromising the later analysis of hand movements.

Using the motion/depth footage of the arms and hands movements ultimately adds another ergonomic variable that can be used in the surgical skills assessment which the dVlogger cannot, since it only records coordinates of the instruments, and does not provide any direct data, visual or numeric, of the arm movements and ergonomic position of the surgeons. Also special to 3D cameras are human pose and skeleton recognition and tracking SDKs, which have demonstrated real-time tracking of human movements [36, 37]. These types of data can be combined with endoscopic footage of the procedures thereby adding inputs for a machine learning algorithm, as has been previously demonstrated using deep neural networks for motion analysis [38, 39]. This may be of relevance to better understand the link between surgical events (as observed through endoscopic images) and surgeon behaviors (as observed through tracking of movements). Combining multiple data sources in the analysis of surgery has been discussed in prior works as being the next step for the future of personalized medicine/surgery [40]. The term Surgomics have been used as an overall term describing multiple features of importance in the surgical setting to collectively better patient outcomes [35]. As examples, the surgeon’s expertise and the difficulty of the procedure are of features of importance. Perhaps by analyzing the surgeons’ movements and position a new feature of Surgomics may arise, underlining the importance of ergonomics during surgery [40].

Another limitation is that it can be technically difficult to prepare the current setup described in this method, which consists of components that are not a part of the robotic surgical system. This may be seen as disadvantageous because the method strives to be a general method that can be used and applied by anyone, regardless of technical know-how. Moreover, the technical demands, or the software/hardware components of the setup, may change or get upgraded, making some of the components outdated, or hard to acquire after some time. However, because the method is an external setup, it gives researchers a higher degree of autonomy and freedom with regard to the output data. Also, because the setup is not an integrated part of any robotic surgical system, meaning that it consists of different external components, it may be more easily fitted to different kinds of robotic surgical systems. This may create a common ground for the standardization of data acquisition across different robot surgical platforms.

The goal of the method described in this study along with other methods of prior studies is ultimately to make way for the implementation of AI-based techniques for automated assessments of surgical skills, events, and prediction of outcomes during RAS. With more studies using and annotating with the same ways and methods, larger available data sets with higher quality and variety may be constructed. This can lead to stronger and more reliable networks as are seen with image data sets from other fields of computer vision-based algorithms [20].

## Conclusion

In conclusion, this paper describes a method of collecting, preparing, and annotating images, events, and motion data from a surgical robotic system. The principles outlined can be used to accelerate the development of the high-quality and quantity data sets needed for future machine learning models for automating the assessment of RAS skills and predict surgical outcomes.
